# Preterm infants on high-frequency oscillatory ventilation: electrical impedance tomography during lung recruitment

**DOI:** 10.1038/s41390-025-04173-z

**Published:** 2025-06-04

**Authors:** Tobias Werther, Erik Küng, Lukas Aichhorn, Angelika Berger, Raffaele L. Dellacà, Chiara Veneroni

**Affiliations:** 1 Division of Neonatology, Pediatric Intensive Care and Neuropediatrics, Department of Pediatrics and Adolescent Medicine, Comprehensive Center for Pediatrics, Medical University of Vienna, Vienna, Austria; 2 TechRes Lab, Department of Electronics, Information and Biomedical Engineering (DEIB), Politecnico di Milano University, Milan, Italy

## Abstract

**Background:**

We introduce a novel physiological parameter derived from electrical impedance tomography (EIT) to evaluate oxygenation-guided lung recruitment maneuvers in preterm infants on high-frequency oscillatory ventilation (HFOV).

**Methods:**

In this prospective observational study, EIT was performed during a single, stepwise oxygenation-guided lung recruitment maneuver in extremely preterm infants. At each step of continuous distending pressure (CDP), we calculated the median oscillations in the aerated region (MOR), defined as the median of oscillatory impedance amplitudes within the air-containing region multiplied by the number of pixels in that region. Recruitability was determined by a ≥15% increase in MOR or oxygenation (S/F-ratio) during deflation compared to inflation at any CDP. Gas exchange parameters were compared between lungs identified as recruitable for MOR or oxygenation.

**Results:**

Of the 56 EIT measurements from 47 infants (mean weight 685 ± 140 g) analyzed, 43 lungs were recruitable by oxygenation criteria, but only 23 met recruitability criteria based on MOR. MOR-recruitable maneuvers significantly improved transcutaneous pCO_2_ by 4.8 mmHg, while non-recruitable maneuvers showed no change.

**Conclusions:**

The novel EIT parameter, MOR, helps identify effective lung recruitment maneuvers and detect overdistention in extremely preterm infants on HFOV, offering the potential to distinguish beneficial from harmful maneuvers.

**Impact:**

- We introduced a novel parameter, the median oscillations in aerated lung regions (MOR), derived from electrical impedance tomography (EIT), to evaluate oxygenation-guided lung recruitment in preterm infants on HFOV.
- The MOR parameter helps in identifying effective lung recruitment in terms of gas exchange and detecting overdistention, offering potential to differentiate beneficial from harmful lung recruitment maneuvers.
- This study presents a practical EIT-based parameter to evaluate lung recruitment and overdistention, providing a precise complement to conventional oxygenation metrics.
- The findings could optimize ventilation strategies in extremely preterm infants, potentially reducing lung injury and improving survival without bronchopulmonary dysplasia.

## Introduction

Bronchopulmonary dysplasia (BPD) is still one of the most important complications in prematurely born infants and is associated with severe pulmonary and neurocognitive sequelae.^1,2^ The incidence of BPD remains high, affecting up to 60% of preterm infants born before 26 weeks of gestation.^3–5^ BPD is primarily triggered by ventilation-induced lung injury (VILI) and oxygen exposure.^3,6^ Consequently, strategies to avoid mechanical ventilation and facilitate oxygenation from the first minutes of life have gained importance.^7^ However, despite the use of noninvasive respiratory support, nearly half of the infants born before 28 weeks of gestation require mechanical ventilation within the first week of life.^8,9^

In response, lung protective ventilation strategies have been developed to mitigate lung injuries caused by ventilation. These strategies rely on two key concepts: the open lung principle and tidal volume targeting.^10–14^ Both principles form the foundation of high-frequency oscillatory ventilation (HFOV), an alternative ventilation strategy that minimizes tidal volumes and pressure changes in the distal airways.^11,15–17^ To approach the optimal lung volume during HFOV, lung recruitment maneuvers (LRMs) guided by oxygenation have been introduced.^18,19^ Stepwise variation in the continuous distending pressure (CDP) helps to resolve lung inhomogeneities, particularly at the initiation of HFOV.^20^

However, using oxygenation as the sole guide for lung recruitment presents two major concerns.^21,22^ First, oxygenation can be influenced by hemodynamic changes due to fluctuations in intrathoracic pressure.^23,24^ Second, oxygenation alone is insufficient to detect lung overdistension.^23–30^ Therefore, recruitment maneuvers should not be used solely to improve oxygenation but rather to increase functional lung volume, i.e., the lung volume that contributes to ventilation.^21,30,31^ Various techniques have been proposed to assess regional lung volume in clinical practice.^32,33^ Among these, electrical impedance tomography (EIT) has proven feasible at the bedside, including in preterm infants.^34^ EIT is noninvasive and radiation-free, offering the advantage of real-time, continuous monitoring of regional ventilation.^29,35–38^

Using EIT, Miedema et al. successfully tracked the pressure–volume curve and identified the lower and upper inflection points in the dorsal and ventral lung regions for most infants with respiratory distress syndrome (RDS).^34^ However, this approach requires stable, high-quality EIT data throughout the procedure, along with the application of a wide range of pressures. Recent animal studies have proposed employing EIT during recruitment maneuvers to assess overinflated regions (aerated at end-expiration with low tidal volume), recruited regions (aerated at end-expiration with significant tidal volume), tidally recruited/de-recruited regions (non-aerated but with significant tidal volume), and collapsed regions (non-aerated with negligible tidal volume).^39,40^ At present, an EIT-derived index that is sensitive to both recruitment and overdistention, accommodating heterogeneous ventilation distribution, and robust against noise and electrode displacement, has yet to be developed.

In this study, we aimed to introduce a novel EIT parameter capable of assessing the oscillating lung volume involved in gas exchange and to examine its changes during standardized LRMs in extremely preterm infants on HFOV. We hypothesized that this parameter could differentiate between LRMs that enhance gas exchange and those that do not.

## Methods

This study is a sub-trial of a randomized controlled trial (ClinicalTrials.gov ID: NCT04289324) investigating recruitment maneuvers during HFOV. It was conducted at the neonatal intensive care unit of the Medical University of Vienna, Austria, between March 2020 and October 2023, and received approval from the local ethics committee (EK 1161/2019).^41^

Preterm infants born before 28 weeks of postmenstrual age, without any congenital anomalies of the heart and/or the lungs (as determined by ultrasound and/or fetal magnetic resonance imaging) were eligible. Infants on HFOV were enrolled based on the availability of the study team to perform measurements during a LRM announced by the caregiving physicians. Written informed consent was obtained in advance from the parents or legal guardians.

### Study protocol

#### HFOV and monitoring

The ventilator used in this study was the Acutronic Fabian HFOi (Vyaire, US). The CDP, amplitude, and frequency were set by the caregiving physicians, with the inspiratory to expiratory ratio fixed at 1:2. The HFOV frequency was not altered during the LRMs. Vital signs were continuously monitored during the measurements, including SpO_2_ (Covidien-Nellcor, Boulder, CO, US) transcutaneous pCO_2_ (tcpCO_2_, SenTec Digital Monitor, Therwil, Switzerland, with a probe temperature of 41 °C), heart rate via ECG electrodes (Micro NeoLead, Neotech Products, CA, US), and invasive blood pressure via a peripheral arterial line connected to a pressure transducer (TruWave pressure transducer, Edwards Lifesciences, CA).

#### LRM on HFOV

LRMs were recommended under the conditions described in ref. ^41^. Starting at the current CDP (initial CDP, CDP_in_), the CDP was increased (inflation limb) approximately every 5 min by 2 cmH_2_O or 1 cmH_2_O when the CDP exceeded 20 cmH_2_O. The fraction of inspired oxygen (FiO_2_) was reduced stepwise to maintain SpO_2_ within the predefined target range (88–96% or 90–96% in the presence of pulmonary hypertension requiring medication). The inflation trial ended when SpO_2_ ceased to improve or when FiO_2_ was ≤0.25.

From the maximal CDP (open CDP, CDP_op_), the CDP was gradually decreased (deflation limb) approximately every 5 min by 2 cmH_2_O or 1 cmH_2_O when CDP was lower than CDP_in_ until a sustained SpO2 drop of at least 5% or a SpO_2_ value below 88% indicated that the closing CDP (CDP_cl_) had been reached. The minimum allowed CDP was 5 cmH_2_O. The pressure amplitude was adjusted to maintain tcpCO_2_ within the target range of 35–65 mmHg. CDP_fin_ was defined as CDP_cl_ + 1 or +2 cmH_2_O and set after returning to CDP_op_ or CDP prior to CDP_op_ (re-open CDP, CDP_re-op_).^18^ The time, HFOV settings, HFOV tidal volume (HFO-TV), and all monitoring parameters were recorded before each CDP change.

#### Electrical impedance tomography

For the EIT recordings, the neonatal textile LuMon Belt was applied around the infant’s chest, at the nipple line, and attached to the LuMon Connector, which transmitted the EIT signal to the LuMon Monitor (Sentec, Landquart, Switzerland).^42^ Small electrical currents (3 mA, 198 kHz) were repeatedly injected in rotation, and voltage changes were measured by all electrode pairs (scan rate 50.86 Hz). The GREIT image reconstruction algorithm generated a 32 × 32 matrix of local impedance.^43^ The EIT measurements were reprocessed to disable the built-in 6.7 Hz filter, which had been applied in the real-time version of the LuMon Monitor software (tic-sw: 1.6.6.000, BL 1.3.1).

### Signal processing of the electrical impedance signal

EIT data were imported into MATLAB R2018 (MathWorks Inc., Natick, MA). Following the approach by Miedema et al.,^34^ we manually selected a stable 30-s segment from the summative impedance signal vs. the end of each CDP interval.^34^

To calculate the EIT-based oscillating volume for each CDP step, we applied a narrow bandpass filter centered at the HFOV frequency (±0.2 Hz) to the selected 30-s segments. We then calculated the median of all local maxima and the median of all local minima for each impedance pixel individually, and generated a difference image by subtracting the minima image from the maxima image. The resulting 32 × 32 matrix ΔZosc represented the regional distribution of EIT-based oscillating tidal volume. Following the approach described by Gartner et al.,^44^ this matrix was normalized to body weight and adjusted by the ratio of HFOV amplitude at CPD_in_ to the current CDP. The sum of all entries in ΔZosc serves as a surrogate for the oscillating tidal volume.^44^

#### Median oscillating amplitudes in the aerated lung regions (MOR)

**Fig. 1:**
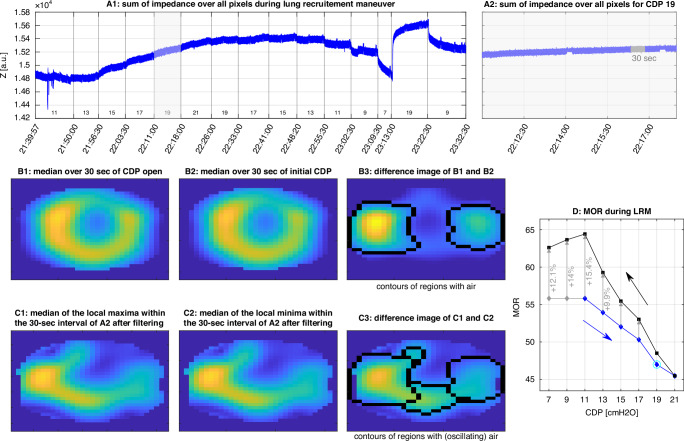
From the EIT measurements to the MOR parameter. **a1** EIT impedance sum signal of an LRM. **a2** The episode of CDP 19 mbar with the manually selected 30-s interval shaded in gray. **b1** Median of the selected 30-s interval at the initial CDP (11 mbar). **b2** Median of the selected 30-s interval at CDP open (21 mbar). **b3** difference image of (**b2**, **b1**) with the black contours surrounding the pixels that exceed the 25% of the maximal value of the difference image. **C1** Median of the local minima within a 30-s interval of (**a2**), filtered using a narrow band-pass filter centered at the HFOV frequency. **c2** Median of local minima of the 30-s interval of (**a2**), filtered using a narrow band-pass filter centered at the HFOV frequency. **c3** Difference image between (**c1**) and (**c2**), illustrating the regional distribution of EIT-based oscillating tidal volume. Contours mark air-containing regions from (**b3**), extended by areas where pixel values exceed 50% of the maximum in (**c3**). **d** MOR for all CDP levels of the lung recruitment maneuver (inflation blue, deflation black). The gray diamonds at CDP levels lower than the initial CDP are shown to illustrate the MOR change used to define recruitability with respect to MOR. The MOR value corresponding to (**c3**) is highlighted with a bright blue circle and amounts to 47.1, calculated as 396 multiplied by 0.119. In this example, the MOR gain between inflation and deflation at the initial CDP is 15.4%, meeting the recruitability criterion for MOR. a.u. arbitrary units, CDP continuous distending pressure, EIT electrical impedance tomography, HFOV high-frequency oscillatory ventilation, LRM lung recruitment maneuver, MOR median of oscillatory impedance amplitudes within the aerated region.

To identify air-containing regions at each CDP step, we first calculated the difference image Δ*Z* as the change between the averaged impedance image (median over 30 s) at CDP_in_ and CDP_op_. Following the approach described by Liu et al., we defined air-containing regions as those pixels exceeding 25% of the maximum Δ*Z* value.^39^ These regions remained fixed across all CDP steps. To ensure that regions with significant oscillations were not excluded, we additionally identified pixels exceeding 50% of the maximum ΔZosc value at each CDP step. We then unified these regions with the previously defined air-containing regions, allowing the combined region to expand with increasing CDP if necessary. Finally, we calculated the median oscillatory impedance amplitude within the unified region and multiplied it by the number of pixels in that region. This resulting parameter, denoted as MOR, incorporates two key features: first, it uses the median of oscillations, thereby penalizing instances where high oscillations in a small region compensate for low oscillations in a larger aerated area—as seen in cases of overdistension (see Fig. S1 in the Supplementary Material), and second, it accounts for pixels in the regions containing (oscillatory) air, thus penalizing collapsed lung areas (see Fig. S4 in the Supplementary Material). We reasoned that this index may be sensitive to the recruitment of functional lung volume. Because it is based largely on relative values of ΔZosc, the MOR parameter is minimally affected by impedance jumps caused by movement artifacts or slight displacements of the EIT belt (Fig. S2 in the Supplementary Material). The complete process, from the raw EIT signal to the calculation of MOR, is illustrated in Fig. 1.

#### Lung recruitability

Gattinoni et al. used a 9% cut-off for recruitable lung tissue to distinguish recruiters from non-recruiters.^45^ In our study, we applied a higher threshold to account for measurement inaccuracies. A lung was considered recruitable if the MOR parameter during deflation exceeded that during inflation by more than 15% at any CDP level of the same magnitude or at the initial CDP of equal or lower magnitude (see example in Fig. 1d). Similarly, for oxygenation, a lung was considered recruitable if the S/F-ratio during deflation exceeded inflation by over 15% for at least one such CDP level.

Using this method, we categorized lungs into four groups: the group with recruitable lungs and the group with non-recruitable lungs, each in terms of oxygenation or MOR. For each group, we calculated the change in oxygenation, tcpCO_2_ (corrected for the current amplitude), HFO tidal volume (corrected for the current amplitude), and the MOR parameter at the following CDP values: CDP_in_ and CDP_in_ + 2 cmH_2_O, CDP_op_−2 cmH_2_O, CDP_op_, CDP^defl^_op_−2 cmH_2_O, CDP^defl^_in_ + 2 cmH_2_O, and CDP^defl^_in_. If the CDP values for inflation did not match those for deflation, linear interpolation was used to estimate all measurements at the appropriate CDP values.

Normally distributed data are presented as means with standard deviations (SDs), while nonparametric data are reported as medians with first and third quartiles (Q1, Q3) or minimum and maximum values (min, max). A Friedman’s repeated-measures rank test, followed by pairwise multiple comparisons using Tukey’s test, was employed to compare data across more than two CDPs, where applicable based on the data distribution. If more than one LRM was recorded for the same participant, these events were treated as independent since they were performed on different days. Consequently, no correction was applied for multiple measurements in the same patient. Differences were considered statistically significant at *p* < 0.05.

## Results

We included 47 preterm infants, from whom we obtained 56 complete measurements during a LRM. The patients’ characteristics are presented in Table 1.

**Table 1:** Baseline characteristics.

Patients (*n* = 47)	
Median PMA at birth (min, max), [wks+d]	24 + 0 (22 + 4, 27 + 6)
Mean weight at birth (SD), [g]	622 (124)
Male, *n* (%)	19 (40%)
Measurements (*n* = 56)	
Median PMA, (min, max), [wks+d]	25 + 1 (23 + 1, 28 + 6)
Median day of life, (min, max), [d]	4.5 (1, 23)
Mean weight (SD), [g]	685 (140)
Mean FiO_2_ (SD) at the start of LRM, [%]	62 (24)
Mean FiO_2_ (SD) at the end of LRM, [%]	44 (21)
LRM with HFOV amplitude changed, *n* (%)	32 (57)

*HFOV* high-frequency oscillatory ventilation, *LRM* lung recruitment maneuvers, *PMA* postmenstrual age, *SD* standard deviation.

**Fig. 2:**
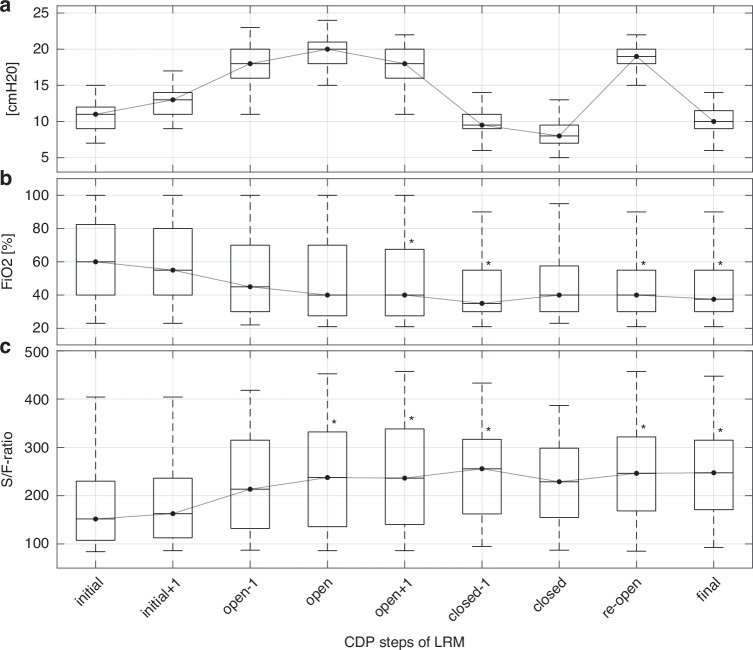
Oxygen-guided stepwise LRM. **a** Boxplots of CDP. **b** Boxplot of FiO_2_. **c** Boxplot of the S/F-ratio. For comparison purposes, only nine CDP steps are shown that were performed for at least every single lung recruitment maneuver. The star close to the upper 75th percentile indicates a *p* value lower than 0.01 in comparison to the initial value (post hoc analysis for the Kruskal–Wallis test). CDP continuous distending pressure, FiO_2_ fraction of inspired oxygen, LRM lung recruitment maneuver, S/F-ratio ratio of peripheral oxygen saturation to FiO_2_.

**Fig. 3:**
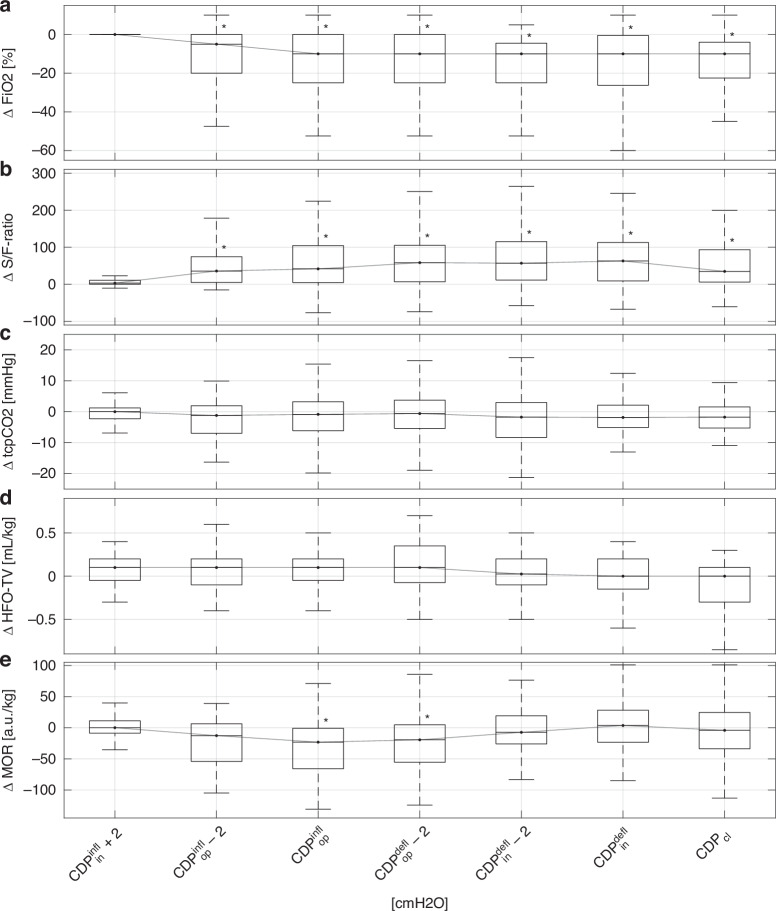
Oxygen-guided stepwise LRM: CDP-dependent change in parameters with respect to the initial value. **a** Boxplots of FiO_2_. **b** Boxplot of the S/F-ratio. **c** Boxplot of tcpCO_2_. **d** Boxplots of HFO tidal volume. **e** Boxplot of MOR. For comparison, initial CDP (+2 cmH_2_O), open CDP (−2 cmH_2_O), and closed CDP were considered for each individual lung recruitment maneuver. In cases where the CDP values did not match the displayed values, a linear interpolation of the values was performed. A star close to the upper 75th percentile indicates a *p* value lower than 0.05 in comparison to the initial value (post hoc analysis for the Kruskal–Wallis test). a.u. arbitrary units, CDP continuous distending pressure, CDP^infl^_in_ initial CDP during the inflation limb, CDP^infl^_op_ open CDP during the inflation limb, CDP^defl^_op_ open CDP during the deflation limb, CDP^defl^_in_ initial CDP during the deflation limb, CDP_cl_ closed CDP, FiO_2_ fraction of inspired oxygen, HFO high-frequency oscillation, LRM lung recruitment maneuver, MOR median of oscillatory impedance amplitudes within the aerated region, S/F-ratio ratio of peripheral oxygen saturation to FiO_2_, tcpCO_2_ transcutaneous partial pressure of carbon dioxide.

The median (min, max) initial, open, closed, re-open, and final CDP values were 11 (7, 15), 20 (13, 24), 8 (5, 13), 19 (11, 24), and 10 (6, 14) cmH_2_O, respectively (Fig. 2). We achieved a significant improvement in oxygenation, with a median (Q1, Q3) increase in the S/F-ratio of 60 (20, 130) during the LRM. No significant changes were observed in tcpCO_2_ and HFO-TV. MOR showed a significant decrease near CDPop (see Fig. 3).

We observed that 43 lungs (76.8%) were recruitable in terms of oxygenation, while 23 lungs (41.1%) were recruitable in terms of MOR. Seventeen lungs (30.4%) were recruitable for both oxygenation and MOR, 26 (46.4%) were recruitable for oxygenation only, 6 (10.7%) were recruitable for MOR only, and 7 lungs (12.5%) were non-recruitable for both oxygenation and MOR. Examples of recruitable and non-recruitable lungs are provided in the Supplementary Material (Fig. S3).

**Fig. 4:**
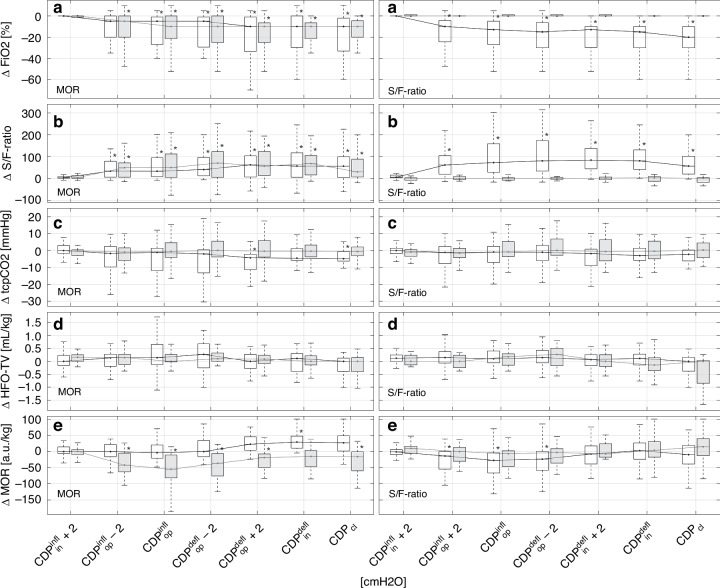
Oxygen-guided stepwise LRM. Left column of illustrations—CDP-dependent changes in parameters relative to the initial value for MOR-recruitable (white boxplot) and MOR-non-recruitable (gray boxplot) lungs. Right column of illustrations—CDP-dependent changes in parameters relative to the initial value for S/F-ratio-recruitable (white boxplot) and S/F-ratio-non-recruitable (gray boxplot) lungs. **a** Boxplots of FiO_2_, **b** boxplots of the S/F-ratio, **c** boxplots of transcutaneous pCO_2_, **d** boxplots of HFO tidal volume, **e** boxplots of MOR. For comparison, CDPin (+2 cmH_2_O), CDPop (−2 cmH_2_O), and CDPcl were considered for each individual lung recruitment maneuver. When the CDP values did not match the displayed values, linear interpolation was performed. A star near the upper 75th percentile indicates a *p* value <0.05 compared to the initial value (post hoc analysis using the Kruskal–Wallis test). a.u. arbitrary units, CDP continuous distending pressure, CDP^infl^_in_ initial CDP during the inflation limb, CDP^infl^_op_ open CDP during the inflation limb, CDP^defl^_op_ open CDP during the deflation limb, CDP^defl^_in_ initial CDP during the deflation limb, CDP_cl_ closed CDP, FiO_2_ fraction of inspired oxygen, HFO high-frequency oscillation, LRM lung recruitment maneuver, MOR median of oscillatory impedance amplitudes within the aerated region, S/F-ratio ratio of peripheral oxygen saturation to FiO_2_, tcpCO_2_ transcutaneous partial pressure of carbon dioxide.

A comparison of the individual changes between the initial value and the inflating and deflating CDP levels for MOR-recruitable lungs, revealed a significant improvement in tcpCO2 (median tpcCO_2_ of −4.8 mmHg between CDP_in_ and CDP_cl_, *p* = 0.039), as shown in Fig. 4 and Table 2. The improvement in tcpCO_2_ was slightly greater in patients with a higher recruitability threshold (15% vs. 30%), see Table 2.

**Table 2:** Comparison of tcpCO_2_ differences between the initial CDP and five different CDP levels at inflation (CDP^infl^_in_ +2 and CDP_op_) and deflation (CDP^defl^_in_ +2, CDP^defl^_in_, and CDP_cl_) for all lung recruitment maneuvers and for recruitable and non-recruitable lungs, where the recruitability cutoff was defined as a 15% (30%) change in the MOR or S/F-ratio, respectively.

CDP level		CDP^infl^_in_+2	CDP_op_	CDP^defl^_in_+2	CDP^defl^_in_	CDP_cl_
Criterion	*N*	tcpCO_2_ difference with CDP initial, median (Q1, Q3), [mmHg]				
ALL	56	0.0 (−2.3, 1.23)	−0.85 (−6.15, 3.23)	−1.75 (8.3. 2.95)	−1.85 (−5.1, 2.1)	−1.75 (−5.25, 1.55)
MOR gain >15%	23	0.0 (−1.7, 3.0)	−1.1 (−11.86, 1.38)	4.3* (−11.35, −2.15)	−4.5 (−5.8, 1.18)	−4.8* (−6.25, −0.6)
MOR gain ≤15%	33	−0.2 (−2.93, 0.43)	−0.5 (−5.91, 1.55)	−0.4 (−3.83, 5.93)	−0.4 (−3.68, 3.23)	−0.6 (−3.13, 2.13)
S/F gain >15%	43	0.0 (−1.8, 1.4)	−0.9 (−7.0, 2.5)	−1.8 (−9.0, 0.8)	−3.0 (−5.8, 1.39)	−2.2 (−6.1, 0.31)
S/F gain≤15%	13	−0.9 (−3.33, 0.5)	−0.8 (−3.5, 5.28)	−0.6 (−6.05, 6.35)	−0.4 (−4.83, 5.53)	−0.3 (−4.25, 4.5)
MOR gain >30%	17	0.5 (−1.98, 3.25)	−2.6 (−13.03, 1.03)	−5.2* (−11.63, −2.63)	−4.8 (−6.08, 1.83)	−4.8 (−5.96, −1.78)
MOR gain ≤30%	39	0.0 (−2.7, 0.48)	−0.7 (−4.46, 3.31)	−0.6 (−4.68, 5.03)	−1.3 (−4.35, 2.75)	−1.4 (−5.21, 2.18)
S/F gain>30%	31	0.0 (−2.7, 1.24)	−1.8 (−10.28, 1.4)	−3.45* (−10.41, 0.35)	−3.3 (−6.75, 0.26)	−3.7* (−7.25, −0.65)
S/F gain≤30%	25	0.0 (−1.98, 0.89)	0.1 (−2.45, 5.28)	−0.65 (−3.93, 6.91)	−0.4 (−4.13, 5.53)	0.3 (−3.3, 3.6)

In cases where the CDP values did not match the displayed values, a linear interpolation of the values was performed.

*CDP* continuous distending pressure, *CDP*^*infl*^_*in*_ initial CDP during the inflation limb, *CDP*_*op*_ open CDP, *CDP*^*defl*^_*in*_ initial CDP during the deflation limb, *CDP*_*cl*_ closed CDP, *Q1* and *Q3* first and third quartiles, *MOR* median of oscillatory impedance amplitudes within the aerated region, *S/F-ratio* ratio of peripheral oxygen saturation to the fraction of inspired oxygen, *tcpCO*_*2*_ transcutaneous partial pressure of carbon dioxide.

*p < 0.05 (post hoc analysis for the Kruskal‒Wallis test in comparison with the value at CDP_in_).

**Fig. 5:**
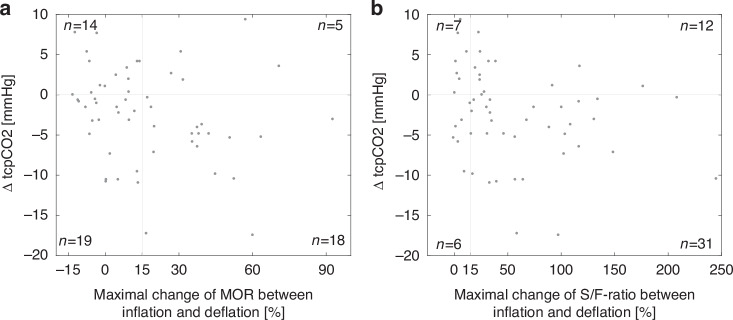
Change in tcpCO_2_ vs. lung recruitment metrics. The difference in tcpCO_2_ (ΔtcpCO_2_) between the initial and closed CDP is shown in relation to the maximal change in MOR (a) and the S/F-ratio (b) for each lung recruitment maneuver. The numbers in the corners represent the number of lung recruitment maneuvers in each quadrant. CDP continuous distending pressure, MOR median of oscillatory impedance amplitudes within the aerated region, S/F-ratio ratio of peripheral oxygen saturation to the fraction of inspired oxygen, tcpCO_2_ transcutaneous partial pressure of carbon dioxide.

When the LRMs were grouped according to the maximum change in MOR between inflation and deflation, a decrease in tcpCO_2_ could be observed in 37 (66%) maneuvers (Fig. 5a). However, in 19 (34%) LRMs no clear improvement in MOR was noted. In no fewer than 14 (25%) cases, LRMs worsened both MOR and tcpCO_2_, suggesting that recruitment likely led to overdistention. In the remaining 5 (9%) cases, MOR improved while tcpCO_2_ increased, possibly indicating overdistention in lung areas not captured by the MOR parameter.

When the LRMs were grouped according to the maximum change in the S/F-ratio between inflation and deflation (Fig. 5b), tcpCO_2_ increased in 19 cases (34%), suggesting that oxygen-guided LRMs may have led to overdistention.

The mean (SD) FiO_2_ and S/F ratio at the start of the LRM for recruitable vs. non-recruitable lungs, based on the MOR parameter, were 61 (23)% vs. 63 (25)% and 176 (88) vs. 174 (78), respectively. For recruitable vs. non-recruitable lungs in terms of oxygenation, the values were 64 (22)% vs. 56 (28)% and 164 (70) vs. 207 (109), respectively. None of these comparisons was statistically significant.

## Discussion

In this prospective observational study, we described a novel EIT parameter (MOR) that quantifies the oscillations in the aerated regions and evaluated its changes in relation to gas exchange during standardized, oxygenation-guided, stepwise LRMs in extremely preterm infants receiving HFOV. We found that LRMs with a gain in MOR significantly improved both oxygenation and CO_2_ removal. Thus, MOR might be helpful to distinguish between effective maneuvers and potentially harmful overdistension, providing valuable insights for managing the open lung concept during HFOV.

The concept of MOR is inspired by the approach of Liu et al.^39^ However, unlike their method, which used impedance values at zero PEEP for image reconstruction and the impedance image at 6 mbar PEEP as a reference, we employed a different approach to identify air-containing regions and additionally incorporated impedance amplitudes to characterize changes in lung function. Costa et al. proposed another EIT method in a decremental PEEP trial, utilizing the best pixel compliance to define percentages of lung collapse and hyperinflation.^46^ The best pixel compliance can only be determined at the end of the PEEP trial. In contrast, MOR is simple to calculate, relying solely on impedance values from the initial, the current and the open CDP step, and can be readily implemented in any EIT system. Furthermore, MOR is robust against motion artifacts and impedance jumps, such as those that occur when the EIT belt needs to be repositioned (see Fig. S2 in the Supplementary Material). Given that EIT systems can now be easily utilized in very small premature babies, this parameter has the potential for clinical application.

MOR was designed to follow changes in functional lung volume during LRMs. It decreased when the maximal CDP was reached, signaling lung overdistension. In lungs categorized as non-recruitable with respect to MOR, we observed a significant decrease in MOR at high pressure levels. Although these lungs exhibited improved oxygenation, CO_2_ elimination did not improve, suggesting that the recruitment may have been detrimental due to overdistention, as indicated by EIT but not reflected in the S/F-ratio (see Fig. 3). In this context, MOR could serve as a valuable complementary tool alongside oxygenation for assessing LRM, particularly given oxygenation’s limitations in detecting overdistension. However, further investigation is needed to determine whether utilizing MOR for lung recruitment during HFOV can help reduce VILI and, more specifically, lower the risk of BPD in extremely preterm infants.

As expected from the nature of the recruitment maneuver, oxygenation improved on average, consistent with its role as the guiding parameter in this approach. This finding aligns with the seminal study by de Jaegere et al.^18^ However, aside from oxygenation, none of the other parameters showed significant changes. Tingay et al. demonstrated that tcpCO_2_ dropped when CDP was decreased during a stepwise recruitment maneuver in HFO-ventilated term or near-term infants receiving muscle relaxants.^22^ We attribute the discrepancy between our findings and those of Tingay et al. to the fact that a substantial proportion of the lungs in our study may not have been recruitable. When we limited the analysis to lungs categorized as recruitable based on oxygenation, tcpCO_2_ actually decreased during deflation. This was also the case when we used MOR to classify lung recruitability. Raising the MOR threshold percentage to tighten the recruitability criterion appeared to further improve tcpCO_2_, similar to the effect seen with oxygenation. This observation highlights the potential of the MOR parameter in assessing lung recruitability, particularly in identifying lungs that respond to recruitment maneuvers with enhanced CO_2_ elimination.

A decrease in pCO_2_ during oxygenation-guided lung recruitment indicates a reduction in dead-space fraction.^21,47^ This leads to two possible interpretations. First, during LRMs where tcpCO_2_ increases, the lungs may either be non-recruitable or hyperinflated. Second, oxygenation may only be adequate for assessing lung recruitment when a significant increase in the S/F-ratio is expected, such as in cases of severe RDS.^18^ Therefore, tcpCO_2_ should be considered an additional parameter for evaluating LRMs as previously suggested by Tingay et al.^22^ However, it should be noted that in five cases classified as recruitable according to MOR, no improvement in tcpCO2 was observed. This could be because the MOR only accounts for the lung region covered by the EIT belt, or due to altered peripheral perfusion affecting tcpCO2 as a result of the hemodynamic changes induced by the recruitment maneuvers. In 19 LRMs, no clear improvement in MOR was observed, despite an improvement in tcpCO_2_. We assume that in these cases, the MOR—limited to the region of the EIT belt—could not reflect improvements in lung areas outside the belt’s range.

Transcutaneous pCO_2_ measurements show a moderate correlation with blood gas CO_2_ in premature infants weighing <1000 g.^48^ Additionally, tcpCO_2_ responds to changes in PaCO_2_ with a delay, meaning that waiting for stable values could prolong overinflation. Patient-initiated breathing can further influence tcpCO_2_, making it challenging to use as a direct indicator of lung mechanics. Meanwhile, SpO_2_ has limitations in detecting overdistention. The MOR parameter may help address some of these challenges. Although it does not represent the entire lung, we believe it sufficiently reflects major lung regions to guide LRM effectively. However, these assumptions require validation in future clinical trials.

In contrast to tcpCO_2_, we did not observe any significant changes in HFOV tidal volume in the subgroup analysis. This may be because changes in HFOV tidal volume at high frequencies are small at airway opening and beyond the resolution of the flow sensor.

In the future, LRM should be refined in two ways: First, we should consider parameters and clinical information to determine whether an LRM is likely to improve lung mechanics and thus justify its application. Second, LRM guidance should be based not only on oxygenation but also on other variables, such as tcpCO_2_ and EIT parameters. Both approaches need to be validated in clinical studies.

### Limitations

This study has several limitations. First, the time intervals for the CDP steps varied, which could introduce recruitment bias. Additionally, the duration of some CDP steps, determined solely by oxygenation, may have been too short to achieve lung volume stability, particularly in heterogeneous lung diseases.^49^ Second, some infants breathed spontaneously during HFOV, while others were administered muscle relaxants. We hypothesize that sedation and muscle paralysis may influence the effects of lung recruitment. Third, HFOV settings, particularly the HFO frequency, were not consistent across all patients. The impact of different HFO frequencies on the efficacy of LRMs remains unknown. Fourth, the definition of lung recruitability is based primarily on oxygenation observations, but limited data support this concept. Fifth, tcpCO_2_ is a parameter influenced by peripheral perfusion and, therefore, does not solely reflect ventilatory aspects.^50^ Lastly, the EIT parameter only refers to a transverse scan of the lung and does not represent the entire lung.

## Conclusion

Our study showed that regional lung data from EIT can track oscillations in aerated lung regions during recruitment maneuvers in preterm infants. The MOR parameter derived from EIT may offer insights into lung recruitment alongside oxygenation, potentially aiding in the management of regional hyperinflation and lung collapse during HFOV. Further research is needed to develop lung protection strategies that incorporate guidance from median impedance oscillations in aerated regions during recruitment.

## Supplementary information

Supplementary Information

## Data Availability

The data will be made available from the corresponding author on reasonable request.
