# Supplementary material for: Preterm infants on high-frequency oscillatory ventilation: electrical impedance tomography during lung recruitment
Source: Pediatr Res. 2025 Jun 4;98(6):2240–8. doi: 10.1038/s41390-025-04173-z (PMC12811120; doi:10.1038/s41390-025-04173-z)
Supplement: Supplementary file 1 — Supplementary Information [file 41390_2025_4173_MOESM1_ESM.pdf]

**Supplementary Material**

# Preterm infants on high-frequency oscillatory ventilation: electrical impedance tomography during lung recruitment

## *1. MOR penalizing instances with high oscillations in a small region*

In the initial approach to defining an EIT-derived parameter for assessing functional lung volume during HFOV, we summed the oscillating impedance (ΔZosc) across all pixels in the region containing air and sufficiently large oscillations (compare black contours in Fig. 1). This value represents the oscillatory tidal of volume this region. However, if there is a small segment with (very) large oscillating impedance values and a large segment with (very) small oscillating impedance values within this region (e.g. in an over-inflated segment), then the sum of the oscillating impedance values could be large, which would result in a misleadingly favorable value. By replacing the sum with the median oscillatory impedance across all pixels in the aerated region (MOR), multiplied by the number of pixels in that region, this issue is resolved. This approach substitutes the influence of high oscillating impedance in a small segment with the median value across all pixels, providing a more realistic and balanced representation of the lung function. We illustrate this phenomenon in Fig. S1.

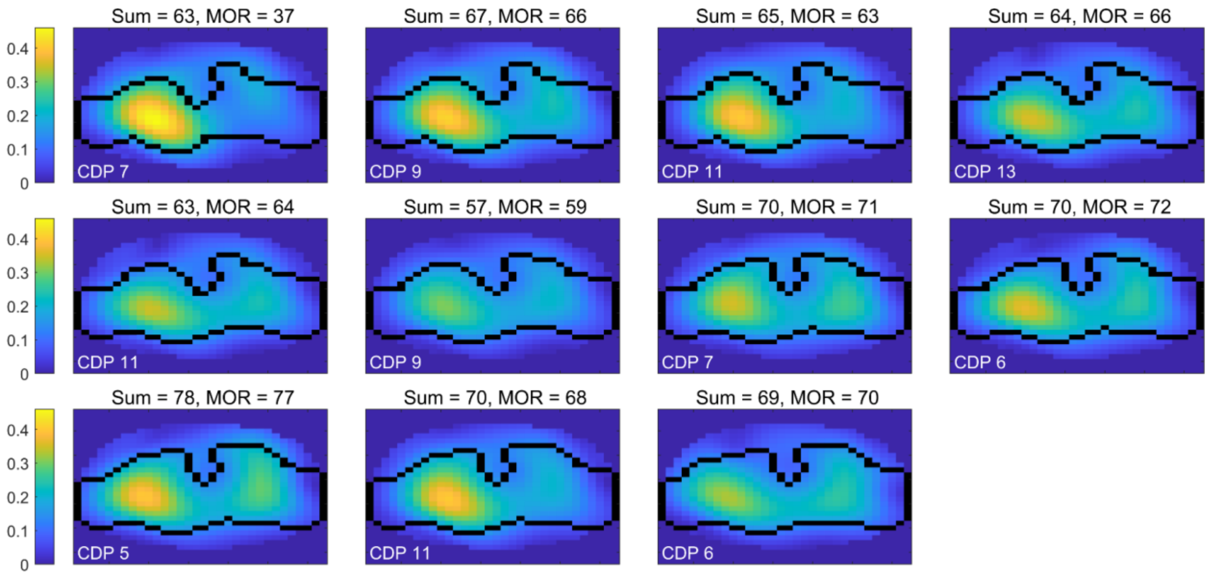

**Figure S1:** Example of the ΔZosc progression with corresponding aerated region contours (in black) during a lung recruitment maneuver performed in an extremely preterm infant (postmenstrual age 24+4 weeks, 3rd day of life, body weight 710 g). The images are oriented with the anterior side at the top and the left side of the body positioned on the right in each image. The area surrounded by the contours defines the aerated region. The first row represents the inflation limb, the second row the deflation limb, and the third row the closing, re-opening, and final CDP. The respective CDP value in cmH2O is displayed in the bottom left-hand corner. At the top of each ΔZosc image, the sum of the oscillating impedance across all pixels (Sum) and the MOR value are displayed. Notably, in the initial CDP, oscillations are predominantly confined to the right dorsal segment, contributing significantly to the sum of oscillating impedance. As a result, the sum is higher compared to the final CDP, where oscillations are more evenly distributed, indicating improved lung function. However, when the MOR value is used instead of the sum of oscillating impedances, an opposite trend is observed: the MOR value in the initial CDP is lower than in the final CDP, reflecting the recruitment of functional lung volume, as evidenced by the more evenly distributed colors. CDP continuous distending pressure, MOR median of oscillatory impedance amplitudes within the aerated region.

### 2. *MOR is resilient to motion artifacts and impedance jumps*

The following example from our data shows impedance signals containing several jumps, likely caused by movement artifacts. These jumps are characterized by a sudden change in the impedance signal (see the light blue vertical lines in Panel A of Fig. S2) and can be corrected (see Panels A and B of Fig. S2). The MOR of the corrected impedance signal differs only slightly from that of the original impedance signal (see Panel C in Fig. S2).

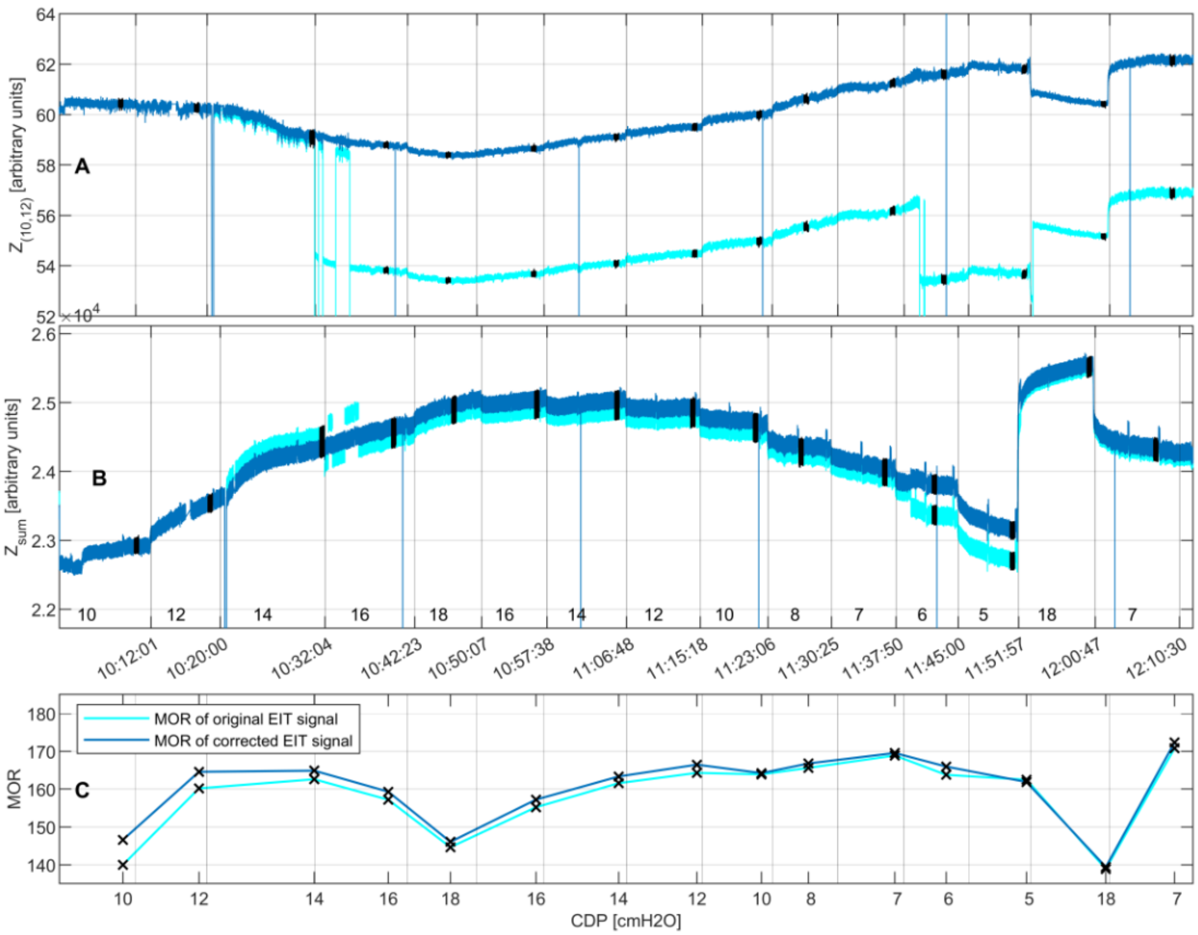


**Figure S2:** Example of impedance signals of a lung recruitment maneuver in a preterm infant (postmenstrual age 28+3, 4$^{th}$ day of life, body weight 470 g) and the corresponding values of median oscillations in aerated regions (MOR). **A**: Original impedance signal with jumps (light blue) and the corrected (jumps removed) impedance signal (dark blue) of a single pixel at the entry (10,12) of the 32x32 matrix. The black parts highlight the selected 30-second signals that were further processed to calculate the MOR values. **B:** The jumps in the original signal are also present in the sum of all impedance signals (light blue) and can be corrected (dark blue). The numbers at the bottom indicate the CDP of the recruitment maneuver. **C:** The MOR values of the original (light blue) and corrected (dark blue) signals show only minor differences, indicating that MOR is largely robust to impedance jumps. CDP continuous distending pressure, EIT electrical impedance tomography, MOR median of oscillatory impedance amplitudes within the aerated region.

## *3. Examples*

### *3.1. Successful lung recruitment*

The following data from stepwise oxygenation-guided lung recruitment in a study participant (postmenstrual age 23+5 weeks, 8th day of life, body weight 705 g) demonstrate improvements in both the gas exchange and MOR parameters. Notably, the MOR parameter at the open CDP is reduced, indicating overdistension, which resolves during deflation and ultimately results in an improved oscillatory impedance signal in the left lung. Gains in both the S/F ratio and the MOR parameter exceeded 15% at the initial CDP level, meeting the recruitability criteria for both oxygenation and MOR.

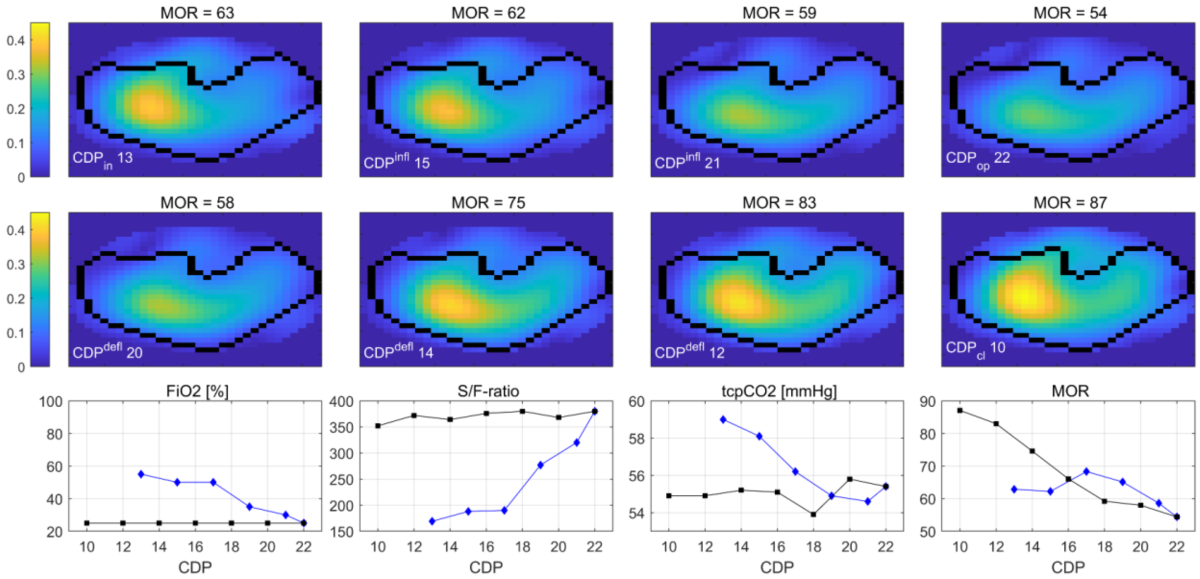


**Figure S3a:** Example of a lung recruitment maneuver. The top row shows ΔZosc with corresponding aerated region contours (in black) during inflation (infl), including the initial CDP (CDPin) and the open CDP (CDPop). The middle row shows the impedance images during deflation (defl), including the closing CDP (CDPcl). The images are oriented with the anterior side at the top and the left side of the body positioned on the right in each image. The area surrounded by the contours defines the aerated region. The bottom row illustrates the progression of FiO2, S/F-ratio, tcpCO2, and MOR across different CDP levels, with inflation

represented in blue and deflation in black. CDP continuous distending pressure, MOR median of oscillatory impedance amplitudes within the aerated region, FiO2 fraction of inspired oxygen, MOR median of oscillatory impedance amplitudes within the aerated region, S/F-ratio ratio of peripheral oxygen saturation to FiO2, tcpCO2 transcutaneous partial pressure of carbon dioxide.

### *3.2. Unsuccessful recruitment in terms of MOR*

In the following stepwise oxygenation-guided lung recruitment in a study participant (postmenstrual age 24+2 weeks, 4th day of life, body weight 625 g) the gain in MOR at each CDP level remained well below 15%, indicating an absence of recruitability in terms of MOR.

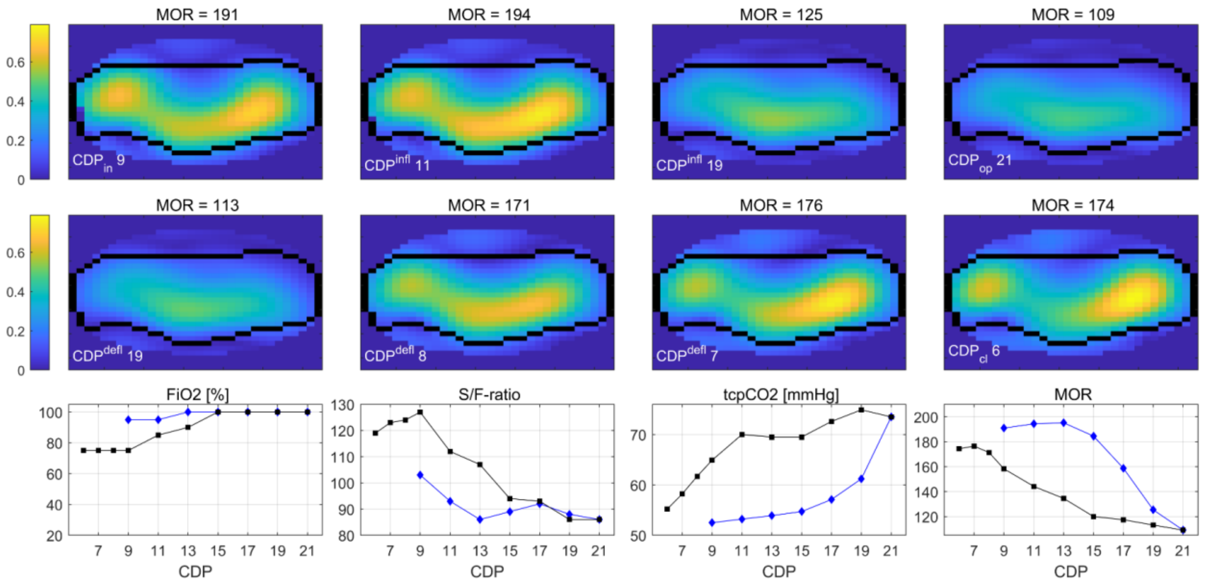


**Figure S3b:** Example of a lung recruitment maneuver. The top row shows ΔZosc with corresponding aerated region contours (in black) during inflation (infl), including the initial CDP (CDPin) and the open CDP (CDPop). The middle row shows the impedance images during deflation (defl), including the closing CDP (CDPcl). The images are oriented with the anterior side at the top and the left side of the body positioned on the right in each image. The area surrounded by the contours defines the aerated region. The bottom row illustrates the progression of FiO2, S/F-ratio, tcpCO2, and MOR across different CDP levels, with inflation

represented in blue and deflation in black. CDP continuous distending pressure, MOR median of oscillatory impedance amplitudes within the aerated region, FiO2 fraction of inspired oxygen, MOR median of oscillatory impedance amplitudes within the aerated region, S/F-ratio ratio of peripheral oxygen saturation to FiO2, tcpCO2 transcutaneous partial pressure of carbon dioxide.

### *3.3. Overdistention*

In the following example of a lung recruitment maneuver in a study participant (postmenstrual age 25+2 weeks, 4th day of life, body weight 710 g), the MOR parameter decreased at the open CDP level, indicating overdistension that persisted throughout the deflation limb. Unlike oxygenation, the tcpCO2 values show no improvement.

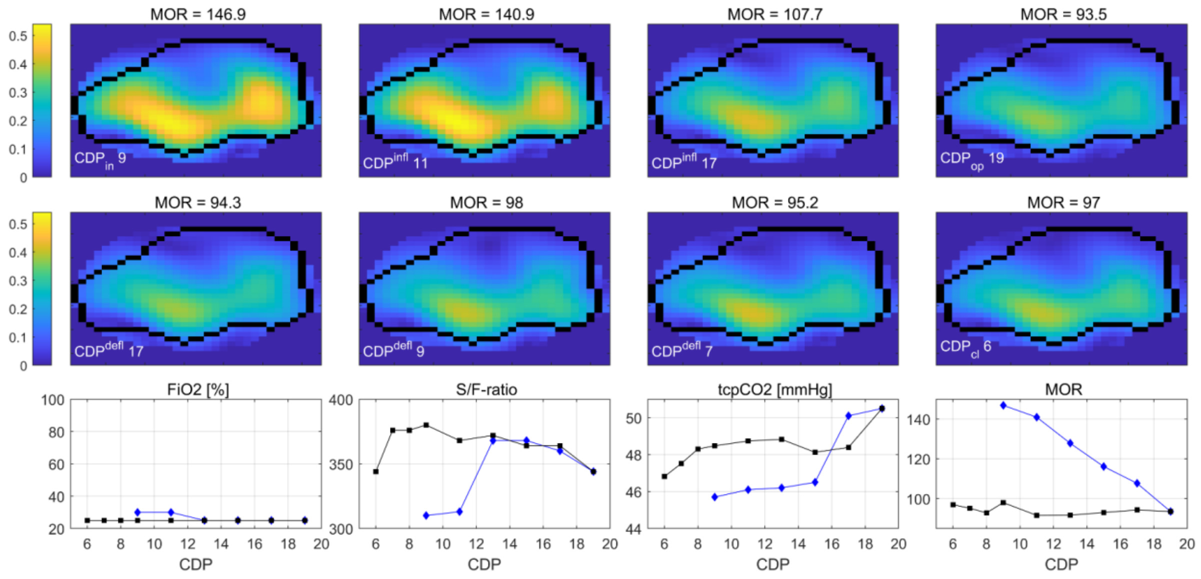


**Figure S3c:** Example of a lung recruitment maneuver. The top row shows ΔZosc with corresponding aerated region contours (in black) during inflation (infl), including the initial CDP (CDPin) and the open CDP (CDPop). The middle row shows the impedance images during deflation (defl), including the closing CDP (CDPcl). The images are oriented with the anterior side at the top and the left side of the body positioned on the right in each image. The area surrounded by the contours defines the aerated region. The bottom row illustrates the

progression of FiO2, S/F-ratio, tcpCO2, and MOR across different CDP levels, with inflation represented in blue and deflation in black. CDP continuous distending pressure, MOR median of oscillatory impedance amplitudes within the aerated region, FiO2 fraction of inspired oxygen, MOR median of oscillatory impedance amplitudes within the aerated region, S/F-ratio ratio of peripheral oxygen saturation to FiO2, tcpCO2 transcutaneous partial pressure of carbon dioxide.

### *3.4. MOR favoring lower CDP levels*

In the following example of a lung recruitment maneuver in a study participant (postmenstrual age 24+2 weeks, 4th day of life, body weight 620 g), the MOR parameter indicated overdistension at the open CDP levels, which resolved during deflation. As the CDP levels were reduced, the MOR parameter continued to improve, favoring levels well below the optimal CDP for oxygenation.

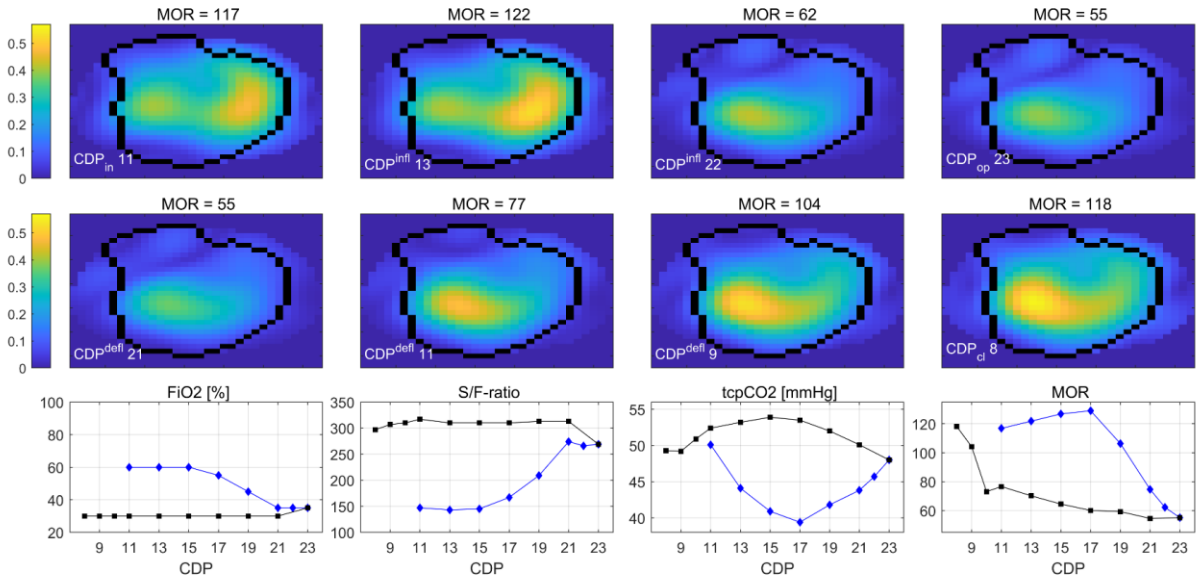


**Figure S3d:** Example of a lung recruitment maneuver. The top row shows ΔZosc with corresponding aerated region contours (in black) during inflation (infl), including the initial CDP (CDPin) and the open CDP (CDPop). The middle row shows the impedance images during deflation (defl), including the closing CDP (CDPcl). The images are oriented with the anterior

side at the top and the left side of the body positioned on the right in each image. The area surrounded by the contours defines the aerated region. The bottom row illustrates the progression of FiO2, S/F-ratio, tcpCO2, and MOR across different CDP levels, with inflation represented in blue and deflation in black. CDP continuous distending pressure, MOR median of oscillatory impedance amplitudes within the aerated region, FiO2 fraction of inspired oxygen, MOR median of oscillatory impedance amplitudes within the aerated region, S/F-ratio ratio of peripheral oxygen saturation to FiO2, tcpCO2 transcutaneous partial pressure of carbon dioxide.

### *3.5. Unsuccessful lung recruitment with a persistent left-right difference*

In the following example, a lung recruitment maneuver in a study participant (postmenstrual age 23+6 weeks, 3rd day of life, weight 550 g) failed to resolve the low oscillatory impedance signal in the left lung, while the right lung exhibited typical overdistension at the open level.

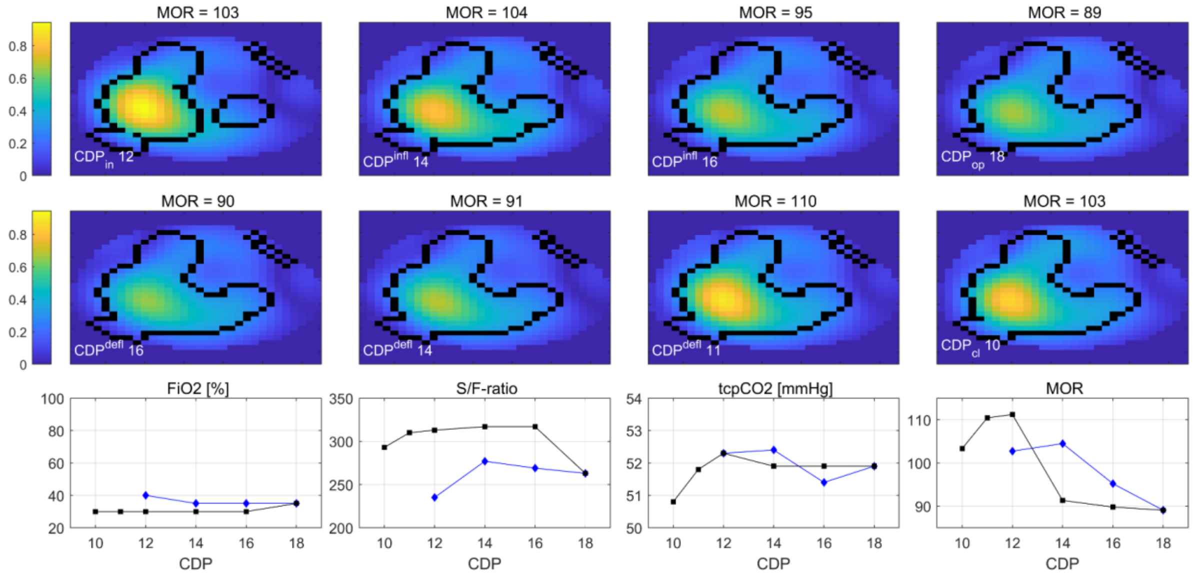


**Figure S3e:** Example of a lung recruitment maneuver. The top row shows ΔZosc with corresponding aerated region contours (in black) during inflation (infl), including the initial CDP (CDPin) and the open CDP (CDPop). The middle row shows the impedance images during deflation (defl), including the closing CDP (CDPcl). The images are oriented with the anterior

side at the top and the left side of the body positioned on the right in each image. The area surrounded by the contours defines the aerated region. The bottom row illustrates the progression of FiO2, S/F-ratio, tcpCO2, and MOR across different CDP levels, with inflation represented in blue and deflation in black. CDP continuous distending pressure, MOR median of oscillatory impedance amplitudes within the aerated region, FiO2 fraction of inspired oxygen, MOR median of oscillatory impedance amplitudes within the aerated region, S/F-ratio ratio of peripheral oxygen saturation to FiO2, tcpCO2 transcutaneous partial pressure of carbon dioxide.

### 4. *MOR versus median ΔZosc*

The median of the 32×32 matrix ΔZosc exhibits a similar trend to the MOR parameter during LRM. However, in certain instances, the median ΔZosc and MOR differ in relation to the recruitability criterion of 15%, as shown in Figure S4.

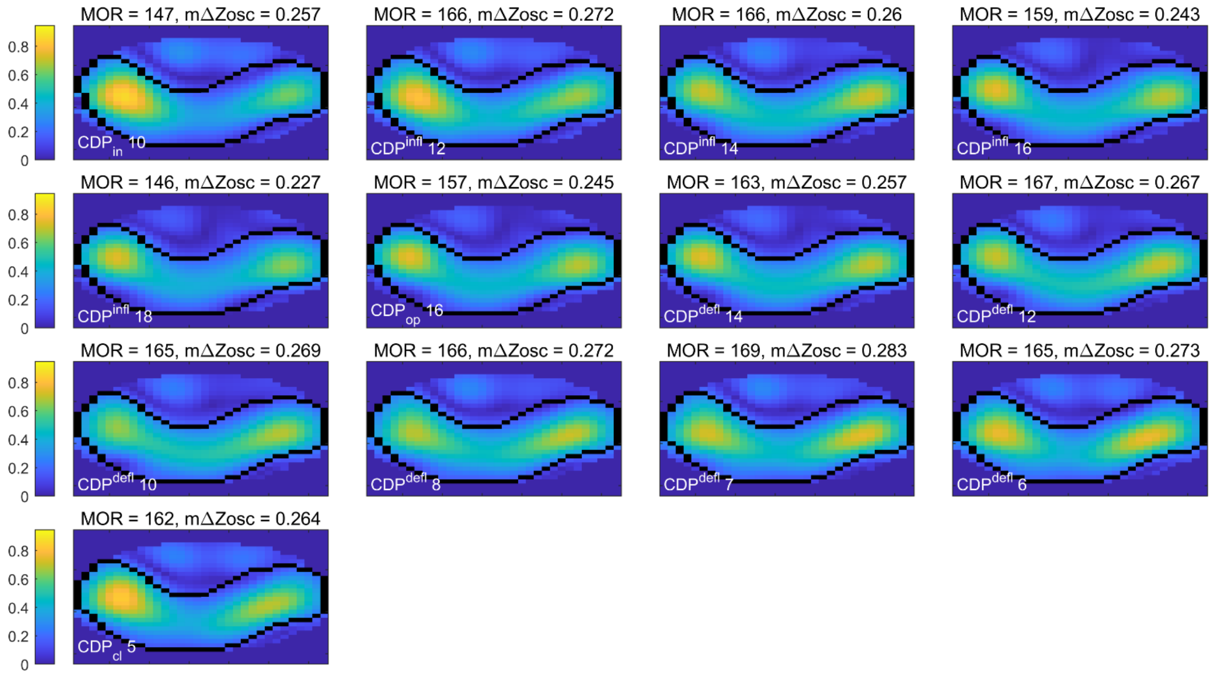


**Figure S4:** Example of a lung recruitment maneuver where the gain in the MOR parameter is higher than that of the median ΔZosc (postmenstrual age 28+3 weeks, 3rd day of life, body weight 470 g, day of life 5, supine position). The images depict oscillating impedance with

corresponding aerated region contours (in black) during inflation (infl) and deflation (defl), including the initial CDP (CDPin), the open CDP (CDPop), and the closed CDP (CDPcl). The images are oriented with the anterior side at the top and the left side of the body positioned on the right in each image. The area surrounded by the contours defines the aerated region. The median ΔZosc is defined as the median ΔZosc over all positive ΔZosc and MOR is defined as the median ΔZosc confined to the aerated region. The maximum gain in MOR between the initial and deflation CDPs is 15.5%, whereas the median ΔZosc shows a maximum gain of 10.3%. CDP continuous distending pressure, mΔZosc median ΔZosc, MOR median of oscillatory impedance amplitudes within the aerated region.
